# Supplementary material for: Controlling a simple model of bipedal walking to adapt to a wide range of target step lengths and step frequencies
Source: PLoS One. 2025 Dec 26;20(12):e0338501. doi: 10.1371/journal.pone.0338501 (PMC12742788; doi:10.1371/journal.pone.0338501)
Supplement: Attachment — Submitted filename: Rebuttal Letter.docx [file pone.0338501.s002.docx]

**PONE-D-25-37501: Controlling a simple model of bipedal walking to adapt to a wide range of target step lengths and step frequencies.**

Dear Professor Martin,

We are pleased to submit the revised version of our manuscript entitled “Controlling a simple model of bipedal walking to adapt to a wide range of target step lengths and step frequencies.”

We sincerely thank you and the reviewers for the thoughtful and constructive feedback. We have carefully addressed all reviewer comments and implemented their suggestions throughout the manuscript. These revisions have improved the clarity, rigor, and overall presentation of the paper.

Please see, in the following, the point-by-point response to all reviewers’ comments and the edits made to the manuscript. We have also attached a revised version of the manuscript implementing all the changes reviewers suggested.

Thank you for considering our revised submission. We appreciate the opportunity to improve our work and look forward to your response.

Sincerely,
Sina Mehdizadeh
(on behalf of all co-authors)

# Reviewer #1:

**PONE-D-25-37501 Review**

Thank you for the opportunity to review this interesting paper.

Here are some comments about the content of the manuscript.

1. Model. I feel that some analytical work on your model 1a,1b,1c can be done, instead of relying only on numerics. At the end, the angle swept during stance should be relatively easy to compute especially under some parameter regimes and this should be enough to determine, at least, an approximate apex return map for the model. See for example what is done in these papers:

- Geyer H, Seyfarth A, Blickhan R. Spring-mass running: simple approximate solution and application to gait stability. J Theor Biol. 2005 Feb 7;232(3):315-28. doi: 10.1016/j.jtbi.2004.08.015. PMID: 15572057.
- Selvitella AM, Foster KL. An approximate solution of the SLIP model under the regime of linear angular dynamics during stance and the stability of symmetric periodic running gaits. J Theor Biol. 2024 Dec 7;595:111934. doi: 10.1016/j.jtbi.2024.111934. Epub 2024 Sep 4. PMID: 39241821.
- Dhawale N, Mandre S, Venkadesan M. 2019 Dynamics and stability of running on rough terrains. R. Soc. open sci. 6: 181729. http://dx.doi.org/10.1098/rsos.181729
- These are simpler or different models, but maybe at least a comment on why finding analytical or approximate solutions is a further challenge wrt what you are addressing in the paper would be useful. It feels to me this might also help your struggles in finding limit cycles numerically, as at least you can guess where parameters for stability are more easily.

Authors’ answer:

We thank you for this insightful comment and fully agree that approximate analytical treatments—such as those developed for the SLIP model—offer valuable intuition and can serve as useful tools to guide numerical work. Kuo’s Idealized Simple Model (Kuo, 2001) is another example, where analytical predictions based on linearization and other simplifications provide important insights.

Similarly, our model could be linearized in the regime of small stance angles, which opens the possibility for deriving closed-form solutions under those assumptions. These analytical approximations could, in turn, be used to generate feasible initial guesses for subsequent numerical root-finding procedures. However, given the wide range of gait parameters we explored—including combinations with large stance angles—such approximations would not be valid or feasible across the entire parameter space.

To acknowledge and incorporate this perspective, we have added the following paragraph to the Methods section:

Line 162:

“While we used numerical approaches to find the limit-cycle gaits, approximate analytical treatments of locomotion models—such as those developed for the Simple Linear Inverted Pendulum model (Geyer et al., 2005; Selvitella & Foster, 2024; Dhawale et al., 2019) or Kuo’s Idealized Simple Model (Kuo, 2001)— could provide valuable intuition and can guide numerical analyses. In principle, our model could also be linearized in the regime of small stance angles, enabling approximate return maps that could generate initial guesses for numerical root-finding. However, because our study spans a wide gait space that includes large stance angles and strongly coupled swing–stance dynamics, such approximations would not remain valid across all conditions. For this reason, we relied on numerical methods to ensure comprehensive coverage of the full gait space.”

2. Stability. Your result in Figure 5 is not surprising, but there is something I am confused about. Depending on the controller, you should be able to cover all the parameter space with stable regions, or not? I think a comment on this early on would make your Figure 5 experiment a little more meaningful, otherwise I do not completely understand the use of it.

Authors’ answer:

To your question: “Depending on the controller, you should be able to cover all the parameter space with stable regions, or not?”—the answer depends primarily on the actuation scheme. With only push-off and a single hip spring, controllability breaks down along the gait space’s uncontrollable manifold, and no controller—regardless of its design—can stabilize those gaits. However, adding a second hip spring restores full controllability across the entire gait space. That said, controllability alone does not guarantee large or useful basins of attraction. One key result illustrated in Figure 5 is that the size of these basins is significantly increased at each grid point, which is essential for the success of our interpolation-based control policy. In the revised manuscript, we have clarified these distinctions and explicitly addressed this point in the new lines below. These additions also respond to related concerns raised by Reviewer 3.

Line 519:

“Our results on stability after control (both maximum tolerable perturbation and the tracking performance) are based on using a linear deadbeat controller. Alternative controller design approaches—such as linear quadratic regulation (LQR), nonlinear controller design, or optimization-based methods—could produce different outcomes. We chose the deadbeat approach because it enables the walker to eliminate disturbances in a single step and avoids the computational overhead associated with some of these other methods. Further, our preliminary experiments did not reveal significant performance differences between the deadbeat controller and an LQR-based controller. Importantly, our design approach—offline computation of controller gains combined with online interpolation—is independent of the specific controller type and can be extended to incorporate other control strategies.”

3. Feedback Policy. Why as an evaluation method you used the root mean square error between the tracked path and the target gait only and did not include the RMSE also of the velocity curves?

Authors’ answer:

We are not sure exactly you mean by “velocity” here. We can think of two types of velocity in this analysis: gait velocity, and derivatives of step length and step frequency at each step. Regarding the former (gait velocity), we should emphasize that there was no explicit target gait velocity to track. However, since the goal of the tracking at each step was to match both the target step length and target step frequency, this ensures tracking of gait velocity because gait velocity = step length X step frequency. Regarding the latter case (the time derivative of step length and step frequency): since the target gait is changing at each step, we wanted to allow the velocity to be whatever it needed to be to accurately track the path.

4. Neural Network Controller. I do not understand this section. I think you jumped from your controller to a feed forward neural network with a not too deep, arbitrary architecture, while there are many other options to compare your controller with other NN ones. It just seems unmotivated and there does not seem to me that there are strong reasons for which this was chosen.

- The way the data is split into training and validation and testing is quite obscure. Of the 90% what percentage is training, what is validation? How was the split determined? Also the number of epochs seems arbitrary and in situation like this, where the loss function is very non convex, I would not be to confident that if you go from 200 epochs to 2000 epochs, the algorithm that seemed to converge to a specific gait does not end up visiting another gait.
- I understand it is tempting to use a NN for this, but there are some cons to consider. First of all, whatever architecture you choose, there is not really a good motivation to support it. I know you mention biologically plausibility, but the network you are using is not more plausible than any others in practice and not even more plausible of more simple msodels. The fact that you save parameters in your architecture, well it’s because you chose that architecture and that policy, but, then, convincingly explaining that a randomly chosen NN architecture with smaller size saves parameters and matches your policy in terms of efficiency is cumbersome and this fact is quite a negative message about your policy.
- The training-validation-test split is quite puzzling no matter what. I know cross-validation might be computationally expensive, but at least you might want to do a second split with the same percentages and similar other two splits with a different percentage in the split (start with one random seed for the first percentage split; then change the seed and repeat). The fact is that CV itself is not theoretically well understood in the iid case and here is so much more complicated that you risk that the true confidence bands of your estimates are enormous.
- At the end, although you are doing your best to be rigorous, you might end up adding a section that damages the paper. It might sound extreme, but there are reasons that make me recommend removing this section (anything NN related) in full.

Authors Answer:

We agree with your concern and useful comments. Therefore, we have removed all the analyses, results, and conclusions related to the neural network policy to keep the coherency of the manuscript as you suggested.

5. Some of your conclusions. Isn’t it already known that open-loop stability is insufficient for gait adaptation? I mean it is already something rare without adaptation involved I would say. I think there needs to be a stronger or more precise conclusion on this, otherwise you might probably benefit from removing this paragraph.

Authors Answer:

You are correct—open-loop stability is not sufficient for gait adaptation, which is precisely why we designed and implemented the feedback controller. However, it was still necessary to document the performance of open-loop stability to then quantify how much adaptation performance increased by adding active control. To do so, we conducted two analyses: one evaluating the maximum tolerable perturbation and another assessing the response time to converge to target gaits.

Although we presented the results of these analyses in the Results section for completeness, we did not emphasize them as primary conclusions in the Discussion section for the very reason you noted. In this regard, we believe our interpretation and your perspective are well aligned with respect to the main contributions of our work.

6. When you add further parameters, you surely increase stability. You made a choice that works, but also this does not seem surprising to me. I think a stronger motivation of why your way is a better way to proceed is definitely needed. What I am trying to say in different words is that you are comparing no-control with control and of course this increases the stability region, basically no matter what type of control you use. Am I confused? If not, I think the motivation and the explanations of why your results are a step forward need to be stronger. And on top of this, there is the issue above of a randomly chosen NN with less parameters performing as efficiently as your policy.

Authors Answer:

Thank you for your thoughtful observation and the opportunity to clarify the motivation and novelty of our approach. We do not believe it is self-evident that simply adding arbitrary control will enhance stability relative to no control; the effectiveness of control depends critically on how it is designed. Moreover, our findings demonstrate that even an optimally designed controller may fail to stabilize the system in certain regions of the gait space if actuation is limited to once-per-step adjustments of push-off and a single hip spring.

Importantly, identifying that stability regions increase with appropriate control is not sufficient to address the more fundamental question we pose: Can this walker adapt at human-like time scales across the entire gait space? Our work directly addresses this question and provides a systematic framework to answer it.

Overall, our work goes beyond this basic no-control/control comparison in at least two key ways:

1. We show that with only a single hip spring and push-off impulse—the conventional actuation used for steady-state walking—it is mathematically impossible to control all gaits in the gait space. Specifically, we demonstrate a zero-boundary where stance angle cannot be influenced, and the controllability matrix becomes rank deficient. This means that no linear feedback scheme—irrespective of gain choice—can stabilize those gaits under the single-spring model. Adding a second independent hip spring is not simply “more parameters,” but the minimal structural change needed to make the system fully controllable across the entire range of human-like gaits. This is a qualitative improvement, not just a quantitative one.
2. Our focus is on whether once-per-step controllers can enable adaptation between diverse gaits within human-like timescales. We show that open-loop stable gaits converge only slowly (hundreds of steps), whereas our deadbeat once-per-step feedback controllers achieve convergence in 2–5 steps for most gaits, closely matching empirical human adaptation studies. Thus, the step forward is not simply expanding the “stability region,” but designing a feedback strategy that enables rapid, robust adaptation across the full gait space using once-per-step control.

We have elaborated these points in both the abstract and first paragraph of the discussion:

Line 14:

“We tested whether the same control principles that support steady-state walking are sufficient for robust, rapid gait adaptation over a wide range of step lengths and frequencies. We begin by demonstrating that periodic gaits exist at combinations of step frequency and step length that span the full range of gaits achievable by humans. However, their open-loop stability is not enough to rapidly transition to target gaits. Next, we show that actuating with only one push-off and one hip spring of fixed stiffness cannot fully control the walker in the entire gait space. We solve this by adding a second hip spring with an independent stiffness to actuate the second half of the swing phase. This allowed us to design local feedback controllers that provided rapid convergence to target gaits by making once-per-step adjustments to control inputs. To adapt to a range of target gaits that vary over time, we interpolated between local controllers. This policy performs well, accurately tracking rapidly varying combinations of target step length and step frequency with human-like response times.”

Line 447:

“We demonstrated that the same method of actuation and control of steady-state walking are insufficient for robust and rapid gait adaptation over a wide range of step lengths and frequencies. Specifically, we showed that relying on only one push-off and one hip spring of fixed stiffness cannot fully control the walker in the entire gait space. Thus, we added a third actuation , allowing the controller to adjust the hip spring stiffness midway through the swing phase. This allowed us to design feedback controllers that stabilized periodic gaits by making once-per-step adjustments to push-off and hip spring stiffnesses providing convergence to target gaits within human-like time scales. Finally, we extended the controller to handle rapidly changing target gaits by interpolating control gains across the grid of periodic gaits.”

# Reviewer #2:

This study investigates the controllability of a simple walking model with one or two simple controllers. It argues that while open-loop control cannot achieve human-like step length and step frequency, it is possible to design a system by adjusting the control inputs for each step. The study comprehensively investigates control performance using the model, and the theoretical development is carefully presented. Overall, the paper is well organized. However, while reading the paper, I encountered a few points that were unclear.

1. One of the main claims of this study is that open-loop control cannot control the entire range of human step length and step frequencies. I agree that this claim is assumed to be correct. The issue is whether this claim is fully supported by the results. I understand that the method used to demonstrate this is to numerically calculate the model's behavior to generate a limit cycle and then analyze the Poincare map around that limit cycle. This limit cycle seems to depend on the initial values, where the paper states that the initial values have been explored through trial and error (lines 152-153). My question is whether this trial-and-error exploration has any effect on the results. Personally, I think that it is probably not a problem in terms of the overall trend, but I have doubts about whether it is possible to make a statement as in line 469, "we showed that ... cannot fully control the ...". If necessary, I would like to see some discussion on whether there is any dependence on the initial values.

Authors’ answer:

We thank the reviewer for raising this important point. The trial-and-error procedure was used only to locate feasible initial guesses for the first gait solution. Once that first solution was identified, we relied on a systematic warm-starting approach to generate the remaining limit cycles across the gait space. We also visually inspected the resulting parameter sets to verify that they varied smoothly across the gait space, with no discontinuities indicating that all solutions belonged to the same family of gaits. Thus, the results do not depend on arbitrary initial values.

We have clarified this point in the revised manuscript:

Line 150:

“We used trial and error to identify feasible initial guesses to find a limit cycle solution for the first gait we solved. This gait had the longest step length and fastest step frequency. We then computed all subsequent gaits systematically using warm-start continuation from neighboring solutions. To ensure consistency, we visually inspected the resulting parameter sets to verify that they varied smoothly across the gait space, with no discontinuities indicating that all of our solutions belonged to the same family of gaits.”

2. The second point concerns the evaluation method for the control system. In "Performance of the feedback policy" in the "Method" section, optimization is used to determine the response time. I found it difficult to understand how this was decided, especially the evaluation function and decision coefficient in lines 258-261, which seemed to appear suddenly. I felt that a more detailed explanation was needed for this part.

Authors’ answer:

We have now clarified this section by providing more detailed explanations.

Line 261-285:

“We evaluated the performance of the feedback policy through two separate analyses. In the first, we computed the optimal response time of the feedback policy as it adapted from perturbed gaits to target gaits. This analysis allowed us to compare the feedback policy’s performance to the response times observed in human walking. To enable this comparison, we aligned our analysis with the experimental design used in empirical studies of human gait adaptation (4,5). That is, for each target gait, we perturbed step lengths 15% above and below the target gait’s step length to specify two perturbed gaits. We then performed an optimization to determine the optimal response time required to converge (i.e. adapt) from the two perturbed gaits to the target gait. For this optimization, we designed two exponential reference paths that shared a time constant to be optimized. These paths began at the perturbed gaits whose step length deviated by ±15% from the target gait and approached the target gait over a pre-specified number of steps (see below). During each iteration of the optimization, the walker tracked these reference paths to converge to the target gait using the feedback policy. The cost function was thus the root mean square error between the actual tracked path and the reference path. The optimization’s decision variable was the exponential time constant of the reference path. In other words, the optimization tried to find an optimal reference path whose tracking with the feedback policy had the lowest root mean square error among all possible reference paths. We repeated this process for perturbations to step frequency as well. As a result, each target gait had two optimal response times, one for step length and one for step frequency, that characterize its convergence speed. We performed this analysis for the five representative target gaits in the gait space described earlier to account for the differences in stability requirements of each region. We allowed a maximum of 10 steps for the reference path length in all target gaits except the target gait in the top left corner which required 30 steps when step frequency was perturbed.”

Minor comments:

3. Line 166: It would be helpful to explain how the disturbance magnitude of 1e-7 was determined. If possible, it would be helpful to know how much influence this 1e-7 has on the motion.

Authors’ answer:

The disturbance magnitude must satisfy two conditions: (1) it should be substantially larger than the numerical tolerance of the integration of the equations of motion—1e-13 in our case—to ensure that its effect is not lost during integration, and (2) it must be small enough for the linear approximations used in Equations 3 and 4 to remain valid.

Our chosen disturbance size of 1e-7 satisfies both conditions. It is sufficiently larger than the integration tolerance, ensuring that numerical eigenvalues are accurate to at least four decimal places. Additionally, our experiments with increasing disturbance sizes showed that the linearity of the return map is preserved up to 1e-3, confirming that 1e-7 lies well within the linear regime. For comparison, Garcia et al. (1998) used an integration tolerance of 1e−12 and a disturbance size of 1e−6. Thus, our selected values fall comfortably within the established range.

We added the following to the paper at line to clarify this:

Line 175:

“We set the perturbation size to 1e-7, which is sufficiently larger than the numerical tolerance of the equation of motion integration (1e-13) to avoid numerical artifacts, yet small enough to preserve the linearity of the return map.”

4. An explanation of the meaning of x in equation (4) would be desirable. I assume that this x is the same as the x in equation (3), but this was not clear when I first read the manuscript.

Authors’ answer:

As the reviewer correctly pointed out, the vector x in equation 4 is the same as in equation 3. We added this now the manuscript:

Line 205:

"... where ${\Delta\bar{x}}_{2\times1}={[\Delta\theta, \Delta\dot{\theta}]}^{T}$similar to Equation (3),…

5. Line 629, Reference 17: The year of publication is missing.

Authors’ answer:

Corrected.

Line 623, Reference 17:

“17.	Zaytsev P, Wolfslag W, Ruina A. The Boundaries of Walking Stability: Viability and Controllability of Simple Models. IEEE Trans Rob. 2018:1–17.”

# Reviewer #3:

The authors investigate simple walking controllers which are sufficient for adapting to various step lengths and frequencies. Their methodology involved: generating a grid of limit-cycle gaits, designing feedback policies for these gaits, and testing two interpolation methods; linear and nonlinear. I find this work methodologically novel and interesting as well as rigorous. I do have some suggestions to further improve the rigor and clarity of the findings below:

1. One notable and surprising finding is the necessity of a third actuation, namely a second torque during the latter half of swing, to achieve control across the full gait pattern space. The paper concludes that a single hip spring and a single push-off are insufficient for such controllability. They show that adding a second hip spring with independent stiffness solves this problem and explains aspects of the swing phase hip muscle activation patterns. Especially because this is an interesting finding, it would be good to test or at least consider alternative explanations or controllers other than a second hip torque: have the authors tested a single spring and damper instead? Can they propose other controllers that may achieve similar stability?

Authors’ answer:

We thank the reviewer for this thoughtful comment and fully agree that alternative actuations, such as a single hip spring combined with damping, are worth exploring. In response to your comment, we added a damper to the single hip spring model and found that it indeed provided control authority over a gait that was previously unstable. This new model has a nice property in that the limit cycle solution still has zero damping and is thus just push-off and a single hip spring actuation. Damping is only needed for stability when perturbed from this limit cycle. This property is shared by our two-hip spring approach which have the same stiffness in the limit cycle and thus function identically to a single hip spring. We believe that there are many other actuation methods that would also provide stability with the unifying property that they allow for independent control of stance and swing dynamics when perturbed from the limit cycle gait. We nevertheless like our method of using two hip springs as it offers a clear biomechanical interpretation—independent hip flexor and extensor torques active in early and late swing. Based on your feedback and suggestions, we now have added discussion of multiple actuation methods, what they have in common, and why we prefer our method to the Discussion section.

Line 492:

“We also explored alternative swing leg actuation strategies beyond the two-spring approach. Specifically, we added a damper in parallel with the single hip spring (38) and found that this modification restored control authority over gaits that were previously unstable. Notably, this configuration has an appealing feature: the limit-cycle solution itself involves zero damping—relying solely on push-off and a single hip spring—while damping becomes active only in response to perturbations. This mirrors the behavior of our two-hip-spring model, where both springs have identical stiffness at the limit cycle and differ only when the walker is perturbed. More generally, a variety of actuation strategies could achieve stability, provided they allow for independent modulation of stance and swing dynamics under perturbation. We favor the two-hip-spring model because it introduces minimal structural complexity while offering a straightforward biomechanical interpretation—corresponding to independent hip flexor and extensor torques active during early and late swing, respectively.”

2. The study also provides an interesting comparison between the two interpolation methods. The neural network based interpolation successfully approximates the control policy but is inferior to the simpler linear interpolation policy in the challenging region of high step lengths. In this region, the neural network either required more steps to complete a path or failed entirely, while the interpolation policy succeeded. This result, while interesting, could be discussed more explicitly and in detail in terms of its biological significance. The authors make an intriguing connection to the findings in Bertram and Ruina, stating “people report difficulty…” at these challenging combinations. Can the authors elaborate what is the nature of this difficulty and how does it relate to the neural network’s difficulties in interpolation?

Authors Answer:

We thank you for this positive comments about the neural network analysis in our manuscript. However, the first reviewer raised some concerns about this analysis and suggested removing this analysis. Therefore, to keep the coherency of the manuscript, we decided to remove this analysis from the manuscript

3. The authors could discuss more prominently how their assumption of a deadbeat and linear controller might affect their findings and conclusions. What if the controller was instead optimized for some other cost function or was a nonlinear feedback control law?

Authors Answer:

We added the following section to the Discussion to account for this comment. These additions also respond to related concerns raised by Reviewer 1.

Line 519:

“Our results on stability after control (both maximum tolerable perturbation and the tracking performance) are based on using a linear deadbeat controller. Alternative controller design approaches—such as linear quadratic regulation (LQR), nonlinear controller design, or optimization-based methods—could produce different outcomes. We chose the deadbeat approach because it enables the walker to eliminate disturbances in a single step and avoids the computational overhead associated with these other methods. Further, our preliminary experiments did not reveal significant performance differences between the deadbeat controller and an LQR-based controller. Importantly, our design approach—offline computation of controller gains combined with online interpolation—is independent of the specific controller type and can be extended to incorporate other control strategies.”

4. Overall, the paper's writing could be more direct. I recommend simplifying complex sentences and using active voice to improve the clarity of the writing. The logical flow of the paragraphs can also be improved.

Authors Answer:

To address your concern, we have reviewed every paragraph with an eye to improve clarity by simplifying the sentences and using more active voice as per your suggestion. We believe it has been improved. Thank you for this suggestion.

5. The methodology for evaluating the feedback policy's tracking performance could be more clearly articulated. While the paper mentions that the walker "starts from the initial gait" to track a path, it does not specify how this initial gait is chosen or if it is varied across different trials. The policy's performance could be dependent on the initial conditions, so clarifying this aspect would strengthen the analysis.

Authors Answer:

We appreciate the reviewer’s question regarding the potential dependence of tracking performance on the location within the gait space. We also shared this concern and therefore selected five representative points across the gait space to evaluate the sinusoidal tracking performance. While this approach does not exhaustively cover all possible gaits, it represents a practical trade-off between computational feasibility and adequate sampling of regions with different stability characteristics. We acknowledge that performance might vary in other parts of the gait space, but we believe our chosen set provides a realistic and informative characterization of controller behavior.

Regarding the initial conditions for the tracking tests, the walker always began at the limit-cycle state of the starting gait. Our rationale was that this reflects a realistic scenario in which a human (or system) begins an adaptation task from an already stable gait, rather than from a perturbed or transient state.

However, we clarified this further in the manuscript:

Line 291:

“As with the convergence rate analysis, we deployed these sine paths in five different regions of the gait space, where each path started from the representative gait as the initial gait. This accounts for the differences in stability requirements of each region. We chose these five points to capture a range of step length–frequency combinations while maintaining computational feasibility. In each case, the walker began from the limit-cycle state of the initial gait, reflecting the realistic scenario in which adaptation begins from an already stable gait rather than from a transient state.”
